# Effects of Abscisic Acid Induction on the Underground Weed Inhibition Strategies of Allelopathic and Non-Allelopathic Rice Accessions

**DOI:** 10.3390/plants14182813

**Published:** 2025-09-09

**Authors:** Jiayu Li, Ting Wang, Xinyi Ye, Shuyu Chen, Yanping Wang, Changxun Fang

**Affiliations:** 1 College of Life Sciences, Fujian Agriculture and Forestry University, Fuzhou 350002, China; lijiayubnu@126.com (J.L.);; 2 College of JunCao Science and Ecology, Fujian Agriculture and Forestry University, Fuzhou 350002, China; 3 Key Laboratory of Crop Ecology and Molecular Physiology, Fujian Agriculture and Forestry University, Fuzhou 350002, China; 4 Fujian Key Laboratory of Agroecological Processing and Safety Monitoring, Fujian Agriculture and Forestry University, Fuzhou 350002, China

**Keywords:** rice allelopathy, abscisic acid, root distribution, rhizosphere microbial community, reversibly adsorbed phenolic acids, benzoic acid derivatives, cinnamic acid derivatives

## Abstract

Despite our preliminary research about the inductive effect of exogenous abscisic acid (ABA) on the weed-suppressive activity of rice in a hydroponic system, there is a lack of knowledge regarding the induction mechanism for ABA application to enhance the ability for weed control underground. Here, two pot experiments using rice–barnyard grass mixed culture were conducted to investigate the effects of exogenous ABA treatment on weed inhibition strategies in both allelopathic rice PI312777 (PI) and non-allelopathic rice Lemont (Le). The largest observed weed inhibition changes in the two rice accessions both occurred with the 9 μmol/L ABA treatment. ABA induction on PI significantly increases the inhibitory effect on the plant height of barnyard grass with root contact and root segregation by 25.7% and 19.1%, respectively, with 23.5% increases observed in Le rice with root contact and no significant increases in plants with root segregation with nylon mesh. ABA induction also significantly increased the root distribution in the soil of Le. Compared with the uninduced group, ABA treatment significantly elevated the total amounts of reversibly adsorbed phenolic acids in the two soil layers of PI and the irreversibly adsorbed phenolic acids in Le soil layers. Furthermore, exogenous ABA could change the bacterial composition in rhizosphere soil of the two rice accessions, with the change in the species composition in the rhizosphere soil of the allelopathic rice PI being greater. Importantly, the bacterial compositions (*Anaerolineales*, *Bacteroidales*, and *Myxococcale*) in the PI rhizosphere soil of rice induced by ABA were more related to the contents of reversibly adsorbed phenolic acids in the soil. However, the core bacterial compositions that promote plant growth (*Sphingomonadales*, *Cyanobacteriales*, and *Rhizobiales*) in the Le rhizosphere soil were more related to the contents of irreversibly adsorbed phenolic acids in the soil. These findings suggested that the ABA induction mainly changed root distribution and core bacterial compositions in Le to enhance resource competition, whereas it stimulated the release of reversibly adsorbed phenolic acids to modulate the specific bacterial compositions in rhizosphere soil of PI and to strengthen allelopathic effects.

## 1. Introduction

Rice allelopathy utilizes specific natural secondary metabolites (allelochemicals) synthesized by rice itself to inhibit weed growth, which is an environmentally friendly and sustainable weed control method that can reduce the use of herbicides and protect the biodiversity of rice fields [1]. It is well known that allelopathic rice releases allelochemicals into the soil through its root, where they have negative effects on surrounding weeds. In particular, the presence of an allelopathic rice root system affected the morphology, nutrient-foraging ability, and competition of the roots of barnyard grass, indicating that allelopathy plays a key role in root recognition behavior during competition between barnyard grass and rice [2,3]. Kong et al. [4] reported that when wheat and heterogeneous plants coexist, chemical interactions occur at the root system level. Wheat can regulate the root distribution pattern of coexisting plants through the release of the allelochemical DIMBOA into the soil. Xu et al. [5] reported that yield increased when the allelopathic variety of rice and its related species coexisted, which was mainly due to the improved distribution of the rice root system and the enhanced competition of allelopathic rice with neighboring weeds, thus compensating for the decreased weed inhibitory effect caused by the decreased amount of allelochemicals. Our previous studies also found that there were significant differences among allelopathic rice and non-allelopathic rice in the root distribution at soil layers, which was significantly correlated with the weed inhibition effect and the contents of allelochemicals [6,7]. These results indicated that modifying the root distribution in soil may be a new method for enhancing the ability of rice plants to protect against adjacent weeds.

The interference of barnyard grass with rice can be mediated by rhizosphere microbial communities. Allelopathic substances released by the rice rhizosphere can cause changes in soil microbes [8]. Studies have shown that allelochemicals secreted by plants can serve as nutrient sources and signaling molecules for soil microbes, thus affecting the composition of soil rhizosphere microbial communities. Similarly, the presence of soil rhizosphere microbes can also affect the composition and secretion of allelochemicals [9,10]. Soil bacteria were notably more responsive to allelochemical addition than fungi, as has been found in previous studies [11,12]. Li et al. [13] reported that the content of phenolic acids secreted by the roots of allelopathic rice was greater than that secreted by the roots of non-allelopathic rice, which led to a significant increase in the number of *M. xanthus* in the rhizosphere soil. In hydroponic planting experiments, some specific phenolic acids secreted from allelopathic rice PI root tissue interact with specific microorganisms (such as *Myxococcus* sp.) in the rice rhizosphere to increase its weed inhibition [14]. Given the importance of soil bacteria in allelopathic interactions between plants, understanding the effect of root exudates in rice rhizosphere soil on the bacterial community is a crucial part of elevating rice allelopathy.

Studies have shown that exogenously applied chemicals, such as salicylic acid, methyl jasmonate, and methyl salicylate, can promote allelopathy in rice [15,16,17]. As the “hidden architect of root system structure” [18], abscisic acid (ABA) has been reported to regulate the root length and development of the allelopathic rice variety Taichung native 1 [19]. In our preliminary test, application of ABA could significantly enhance the weed-suppressive activity of both allelopathic rice and non-allelopathic rice through regulating root growth and the synthesis of rice allelochemicals in a hydroponic system [20]. However, there is still a lack of knowledge regarding the induction mechanism for exogenous ABA to enhance the weed control ability of allelopathic rice and non-allelopathic rice underground. To address this, we adopted an inhibitory-circle method, in which rice accession and barnyard grass were cultured together in paddy soil under natural conditions. We investigated the effects of exogenous ABA treatment on weed inhibition strategies in both allelopathic and non-allelopathic rice. Furthermore, we compared the horizontal distribution patterns of rice roots in paddy soil and phenolic acid content in root exudates of two rice varieties before and after ABA induction. Finally, we focused on the changes in the diversity and community composition of bacterial and microbial taxa in different soil layers under ABA treatment, and additionally analyzed the correlation between the phenolic acid content secreted by rice roots and the bacterial composition in the rhizosphere soil.

## 2. Results

### 2.1. Weed Inhibition Changes Under ABA Induction

**Figure 1:**
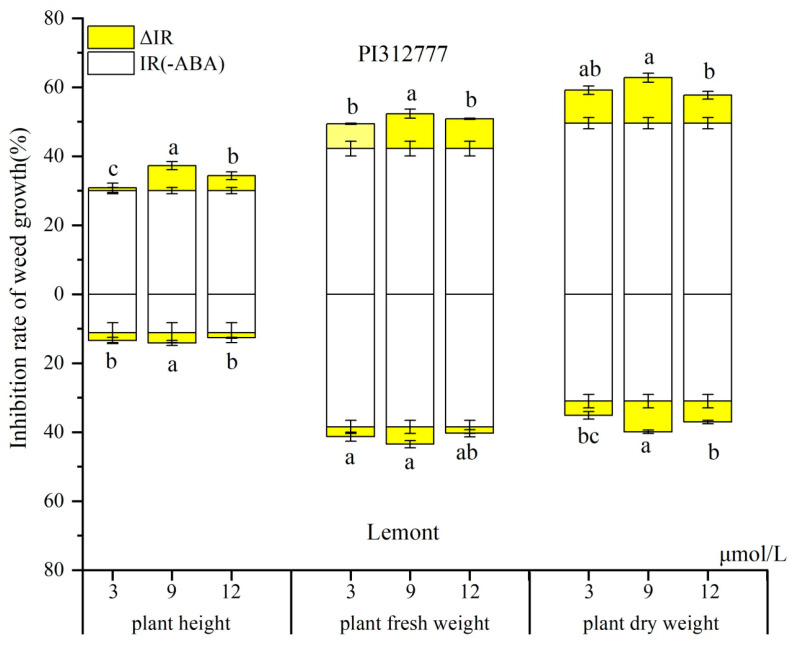
Inhibition of barnyard grass growth by PI and Le rice under induction treatment with exogenous ABA at different concentrations. IR, the % inhibition without ABA induction, ΔIR, increment of the % inhibition with ABA induction. Values plotted are means plus/minus SE. Significant differences (*p* < 0.05) between ABA treatments within the same rice cultivars are indicated by different lowercase letters.

In the pot test, treatment with exogenous ABA at different concentrations significantly elevated the inhibitory effect of the two rice cultivars on the growth of the mix-cultured weeds (Figure 1). Allelopathic rice PI caused a stronger inhibition of the plant height, plant fresh weight, and plant dry weight of the barnyard grass than that of non-allelopathic rice Le. Regardless of the allelopathic traits of the rice cultivars, the largest observed weed inhibition changes occurred with 9 μmol/L ABA induction. Under this optimal induction condition (9 μmol/L ABA), the increment of the inhibition rate (ΔIR) on the plant dry weight of the barnyard grass was 13.2% in PI (increased from 49.5% to 62.8%) and 8.91% in Le (increased from 31.0% to 39.9%). Furthermore, the increment proportions (ΔIR/IR) of PI and Le were 26.7% and 28.7%, respectively, compared with the inhibition rate of rice without ABA induction (IR). Similar results were observed with the increment of the inhibition rate on the plant height and plant fresh weight of the barnyard grass, but the increment proportion (ΔIR/IR) on plant fresh weight with Le was less than that with PI.

**Figure 2:**
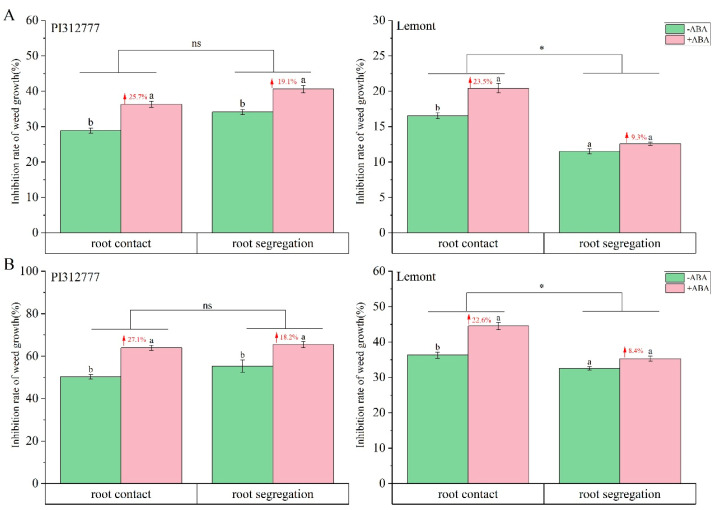
Effect of exogenous ABA on the inhibition of plant height (**A**) and plant dry weight (**B**) of barnyard grass mix-cultured with two rice cultivars during root contact or root segregation with nylon mesh. Values plotted are means plus/minus SE. Different letters above the bars represent significant differences between ABA treatments within the same root treatment (*p* < 0.05, *t*-test). The numbers with red arrows represent the increase in inhibition rate induced by ABA within the same root treatment group. Asterisks indicate significant differences between the root treatments. ns, not significant (two-way ANOVA, * *p* < 0.05).

To further elucidate the impact of exogenous ABA application on the root competition effect and allelopathic effect of two rice cultivars, we investigated the changes in the inhibitory effect on the plant height (Figure 2A) and plant dry weight (Figure 2B) of barnyard grass during two root treatments (root contact or root segregation with nylon mesh). As shown in Figure 2A, compared to the control group without ABA application, ABA induction on PI significantly increased the inhibitory effect on the plant height of barnyard grass with root contact and root segregation by 25.7% and 19.1%, respectively. Although two-way ANOVA revealed that the increases in the inhibition rate levels of the two root treatments were statistically similar, the increases in the inhibition rate observed with the root contact treatment were 6% greater than those observed in root segregation with nylon mesh. Moreover, the enhanced inhibitory effect of ABA for Le rice was not consistent between two root treatments, with 23.5% increases observed in plants with root contact and no significant increases in plants with root segregation with nylon mesh (Figure 2A). Variance analysis in weed inhibition changes of Le showed significant differences between two root treatments. Similar results were also found for the changes in inhibitory effect on plant dry weight of barnyard grass during two root treatments, with no significant differences for PI and significant differences for Le (Figure 2B). The difference between the two root treatment methods was that root segregation with nylon mesh prevented penetration of root systems, which reduces the root competition between rice and barnyard grass in the underground. However, both root contact and root segregation with nylon mesh allowed chemical and microbial interactions in the pots, which was referred as the allelopathic effects of rice on weeds.

Together, these results suggest that the mechanism by which exogenous ABA affected weed inhibition varied in two rice cultivars with different allelopathic potentials. That is, ABA enhances the inhibitory effect of non-allelopathic rice Le on the growth of barnyard grass mainly due to its impact on the competitive effect of rice roots. For allelopathic rice PI, the induction effect of ABA is more related to root exudates and microbial interactions in the soil, coupled with the weak competition of roots.

### 2.2. Effect of ABA on Horizontal Distribution of Roots in Paddy Soil

**Figure 3:**
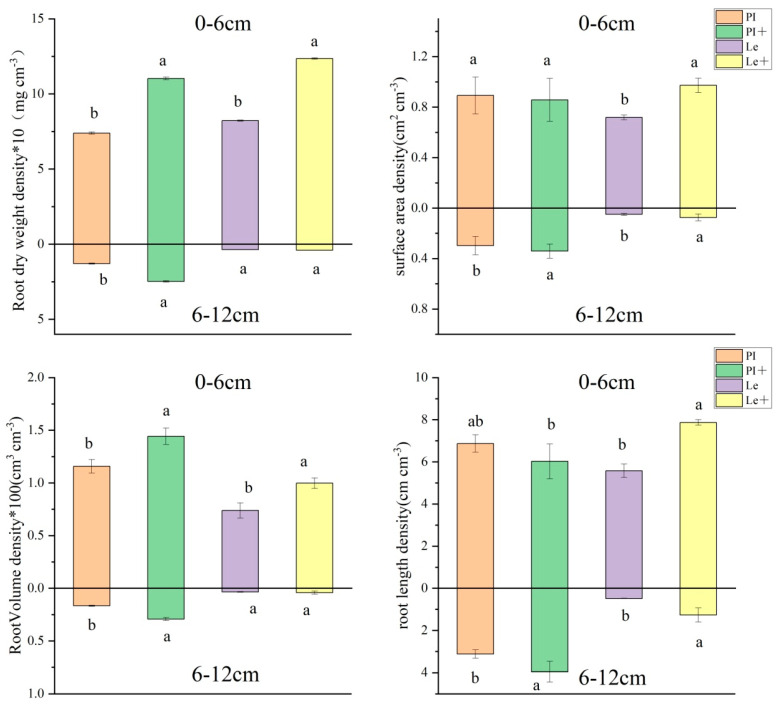
Effect of exogenous ABA on the distribution of rice roots in different soil layers. PI: PI without ABA treatment, PI+: PI with ABA induction, and Le: Le without ABA treatment, Le+: Le with ABA induction. Values plotted are means plus/minus SE. Significant differences (*p* < 0.05) between ABA treatments at the same distance are indicated by different lowercase letters.

The effect of exogenous ABA on the distribution patterns in root length density (RLD), root surface area density (RSD), root volume density (RVD), and root dry weight density (RWD) of two rice cultivars at different horizontal distances at the seedling stages is shown in Figure 3. Under the induction of 9 μmol/L ABA, the RLD, RSD, RVD, and RWD in the 0–6 root zone of Le significantly increased by 41.0%, 35.4%, 35.2%, and 50.3%, respectively, yet only RVD and RWD in the 0–6 root zone of PI increased by 24.7% and 49.4%, respectively. However, four root measures followed a different trend at a distance of 6–12 cm. Highly significant differences were observed in RVD, RWD, RLD, and RSD between the control and ABA treatment groups of PI at 6–12 cm soil circle, with a rise of 75.8%, 91.9%, 26.7%, and 8.1%, respectively, though only the RLD of Le rice root showed a significant enhancement in the 6–12 cm soil layer. These results indicate that exogenous ABA treatment can significantly enhance the root morphology indicators of non-allelopathic rice in the soil, while also promoting more outward growth of allelopathic rice roots.

#### 2.2.1. Effect of Exogenous ABA on the Horizontal Distribution of Rice Allelochemicals in Soil

**Figure 4:**
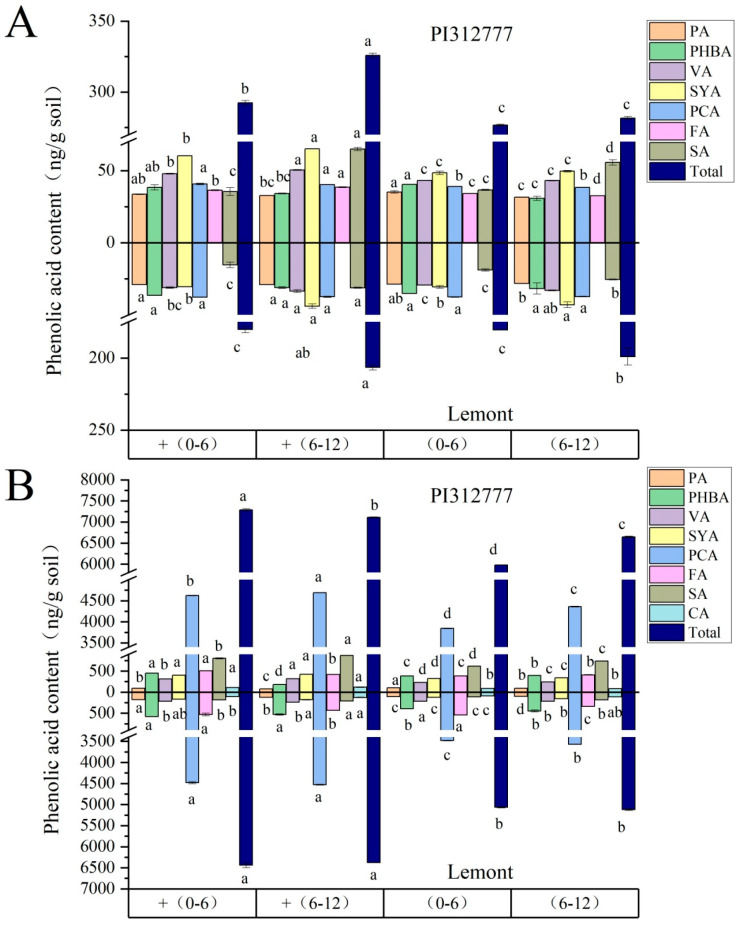
Contents of reversibly adsorbed phenolic acid (**A**) and irreversibly adsorbed phenolic acid (**B**) in rhizosphere soil of different soil layers of two rice cultivars induced by exogenous ABA. The soil samples in the 0–6 and 6–12 cm soil layers of each rice cultivar without ABA induction are denoted as (0–6) and (6–12), respectively, while the root samples of two rice cultivars induced by ABA are denoted as + (0–6) and + (6–12), respectively. PA: protocatechuic acid; PHBA: *p*-hydroxybenzoic acid; VA: vanillic acid; SYA, syringic acid; PCA: *p*-coumaric acid; FA: ferulic acid; SA: salicylic acid; CA: cinnamic acid. Total: Total content of eight single phenolic acids. Lowercase letters indicate significant difference at *p* < 0.05 level between different treatments with the same phenolic acid on the same rice cultivar.

When two rice seedlings were exposed to exogenous ABA, the trends of change in the content of each individual phenolic acid (salicylic acid, vanillic acid, syringic acid, protocatechuic acid, *p*-hydroxybenzoic acid, *p*-coumaric acid, ferulic acid, and cinnamic acid) in different soil layers were not completely consistent, whether for reversibly adsorbed phenolic acid or irreversibly adsorbed phenolic acid (Figure 4). Due to the stronger weed-suppression effect of phenolic acid mixtures compared to each individual phenolic acid, we focused on the changes in total content of phenolic acids (the sum of eight phenolic acids). For the reversibly adsorbed phenolic acids which can be directly utilized by the weed or microorganisms (Figure 4A), the total amounts of reversibly adsorbed phenolic acids in the 0–6 cm and 6–12 cm soil layers of PI were 0.292 mg/g and 0.326 mg/g, respectively, which were significantly greater than those in the uninduced group, increasing by 5.68% and 15.7%, respectively. Nevertheless, ABA treatment resulted in no significant change in the total content of reversibly adsorbed phenolic acids in the two lateral distance soil layers of Le. Additionally, as shown in Figure 4B, the total contents of irreversibly adsorbed phenolic acids in the 0–6 cm and 6–12 cm soil layers of Le were 6.44 mg/g and 6.38 mg/g, respectively, which were significantly greater than those in the untreated control group, with increases of 27.1% and 24.5%, respectively. ABA treatment elevated the irreversibly adsorbed phenolic acids in the two soil layers of PI by 21.9% and 6.98%, respectively.

**Figure 5:**
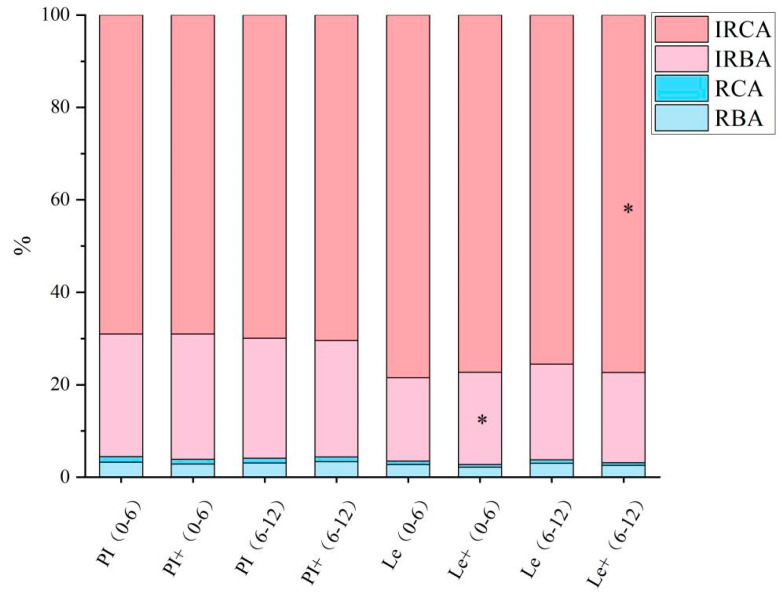
The proportion of different types of phenolic acids in rhizosphere soil of different soil layers of rice induced by exogenous ABA. The soil samples in the 0–6 and 6–12 cm soil layers of each rice cultivar without ABA induction are denoted as (0–6) and (6–12), respectively, while the root samples of the two rice cultivars induced by ABA are denoted as + (0–6) and + (6–12), respectively. RBA: reversibly adsorbed benzoic acid derivatives, RCA: reversibly adsorbed cinnamic acid derivatives, IRBA: irreversibly adsorbed benzoic acid derivatives; IRCA: irreversibly adsorbed cinnamic acid derivatives. Asterisks indicate significant differences between ABA treatments in the 0–6 and 6–12 cm soil layers of the same rice cultivar (* *p* < 0.05).

Based on their chemical structure, the reported phenolic acid compounds with allelopathic potential were mainly classified into benzoic acid derivatives (the content sum of *p*-hydroxybenzoic, syringic, protocatechuic, vanillic, and salicylic acid) and cinnamic acid derivatives (the content sum of ferulic acid, *p*-coumaric, and cinnamic acid). We further compared the proportions of different types of phenolic acids in the total phenolic acid pool at the 0–6 cm and 6–12 cm soil layers of two rice cultivars induced by exogenous ABA (Figure 5). These data showed that the proportion of various phenolic acids in the two soil layers of PI after ABA induction were not significantly different from those in the uninduced group. However, after the induction of Le, the proportion of the contents of irreversibly adsorbed benzoic acid derivatives in the 0–6 cm soil layer significantly increased, and the contents of irreversibly adsorbed cinnamic acid derivatives in the 6–12 cm soil layer also significantly elevated.

#### 2.2.2. Effect of Exogenous ABA on the Bacterial Communities in Different Soil Layers

**Figure 6:**
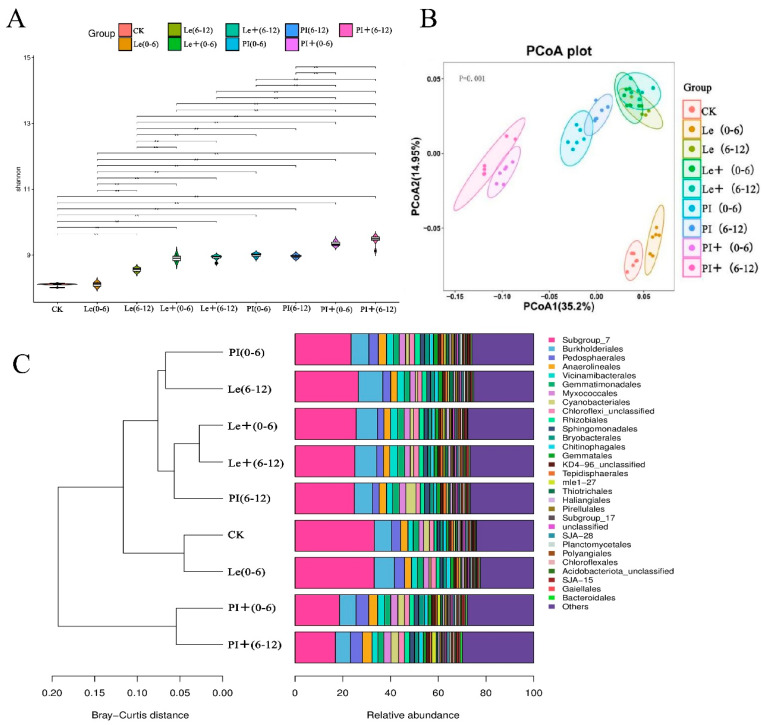
Modulation of the specific bacterial taxa in the 0–6 and 6–12 soil layers of PI and Le induced by exogenous ABA. The soil samples in the 0–6 and 6–12 cm soil layers of each rice cultivar without ABA induction are denoted as (0–6) and (6–12), respectively, while the root samples of the two rice cultivars induced by ABA are denoted as + (0–6) and + (6–12), respectively. CK, monocultures of the barnyard grass. (**A**) The Shannon index of the nine types of soil. ** indicates significant difference at *p* < 0.01 level. (**B**) Principal coordinate analysis (PCoA) of bacterial community of three types of soil based on Bray–Curtis distance. (**C**) Accumulation clustering map of order distribution of the rhizosphere microbiota compositions in the nine types of soil.

At the OTU level, differences in the soil microbiota in the 0–6 and 6–12 soil layers of allelopathic rice PI and non-allelopathic rice Le before and after induction were examined. After ABA induction, compared with that in the uninduced group, the bacterial communities in the soil layers of the two rice varieties differed. Except for the 6–12 soil layers of PI, the specific enrichment of soil bacterial communities in the rice root zones increased (Figure S1). Moreover, the composition of the bacterial microbiota in soil with the addition of ABA differed from that in soil with no treatments (Figure 6). The Shannon indices of the rhizosphere microbiota associated with the two rice varieties increased significantly compared to those of the uninduced group, and the higher diversity of rhizosphere microbiota associated with allelopathic rice PI (Figure 6A), indicating that PI treated by ABA recruited more bacterial species than those of Le. Unconstrained PCoA of Bray–Curtis distance revealed that the soil bacterial microbiota of PI before and after induction gradually separated along the first axis and that of Le gradually separated along the second axis before and after induction (Figure 6B). Furthermore, ABA induction resulted in two rice rhizosphere soils having different abundances of core and unique bacterial microbes. To simplify the statistics, we selected the 30 most abundant microorganisms at the order level and compared the differences in their abundance in the collected samples (Figures S2 and 6C). The ones with the highest abundance were *Subgroup_7* and *Burkholderiales*, which were the lowest in the rhizosphere soil of PI under ABA treatment, whereas the relative abundance of *Pedosphaerales* in bacteria were higher in the rhizosphere soil of PI induced by ABA. Notably, we focused on the relative abundance of *Myxococcales* which were related to rice allelopathy in some studies. In all collected samples, *Myxococcales* were present at higher relative abundances in the 0–6 and 6–12 cm soil layers of the induced PI, accounting for 3.07% and 3.01%, respectively. Additionally, the cluster map again showed that exogenous ABA could change the bacterial composition in the rhizosphere soil of the two rice accessions, with the change in the species composition in the rhizosphere soil of the rice PI being greater.

**Figure 7:**
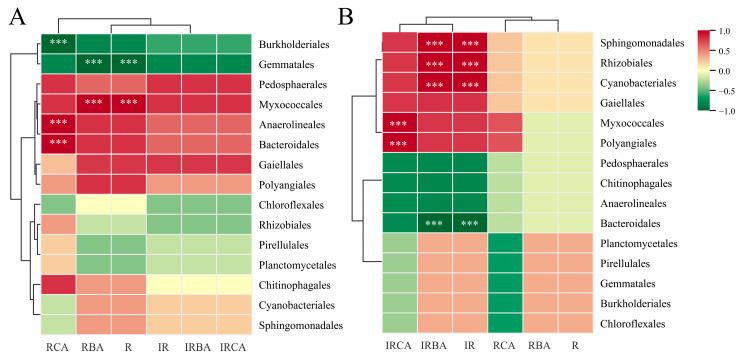
Spearman correlation analysis between bacterial communities and phenolic acids in the rhizosphere soil of PI (**A**) and Le (**B**) induced by exogenous ABA. R: Total reversibly adsorbed phenolic acid; IR: Total irreversibly adsorbed phenolic acid; RBA: reversibly adsorbed benzoic acid derivatives, RCA: reversibly adsorbed cinnamic acid derivatives, IRBA: irreversibly adsorbed benzoic acid derivatives; IRCA: irreversibly adsorbed cinnamic acid derivatives. In Spearman correlation analysis, the corresponding intermediate heat map value is the Spearman correlation coefficient r, r > 0 is the positive correlation, r < 0 is negative correlation. *** indicates the significance test *p* < 0.001.

To investigate the roles of phenolic acids in rice rhizosphere soil in the recruitment of soil bacterial microbiota, we analyzed the relationships between the contents of different types of phenolic acids in soil with the relative abundance of 15 bacterial species, which were screened from the top 30 species at the order level by the Metastat method (Figure 7). Spearman’s analysis showed that the bacterial compositions in the PI rhizosphere soil of rice induced by ABA were more related to the contents of reversibly adsorbed phenolic acids in the soil. Specifically, there was a significant positive correlation between the total amounts of reversibly adsorbed cinnamic acid derivatives and the relative abundance of *Anaerolineales* and *Bacteroidales*, and between reversibly adsorbed benzoic acid derivatives and the relative abundance of *Myxococcale*. *Burkholderiales* and *Gematales* in the bacterial compositions showed significantly negative associations with reversibly adsorbed phenolic acid contents in root exudates (Figure 7A). However, the bacterial compositions in the Le rhizosphere soil of rice induced by ABA were more related to the contents of irreversibly adsorbed phenolic acids in the soil (Figure 7B). The relative abundance of *Sphingomonadales*, *Cyanobacteriales*, and *Rhizobiales* was significantly positively correlated with the total amount of irreversibly adsorbed benzoic acid derivatives in the soil. *Myxococcales* and *Polyangiales* in the bacterial compositions showed significantly positive associations with the contents of irreversibly adsorbed cinnamic acid derivatives in root exudates. These results suggested that the phenolic acid allelochemicals secreted by rice roots into the soil might be involved in the establishment of soil microbial communities under exogenous ABA application.

## 3. Discussion

It was proven that the application of exogenous plant hormones improves weed-suppression ability in hydroponic experiments by regulating rice roots at the seedling stage [21]. Our previous study has shown that exogenous ABA treatment can promote the increase in induction of allelopathy in non-allelopathic rice towards that of allelopathic rice, indicating that rice allelopathy is an induction mechanism [20]. From a model system of rice–barnyard grass mixed culture in paddy soil, we found a consistent result that the proportion of the increased inhibition rate (ΔIR) to the total inhibition rate after ABA induction in non-allelopathic rice Le was not significantly different from that in allelopathic rice (Figure 1), and ABA induction impacted the root length density, root surface area density), root volume density, and root dry weight density of the two rice accessions in two soil layers (Figure 2). However, rice is a field crop, and soil is the natural substrate where rice and weed roots grow [22]. Therefore, it is of more practical significance to explore the regulatory process of exogenous ABA on rice allelopathy by affecting the rice root system in the underground soil.

### 3.1. Different Weed Inhibition Strategies of Allelopathic Rice and Non-Allelopathic Rice After ABA Induction

The competition for resources and allelopathic effects between plants often coexist, and distinguishing between rice resource competition and allelopathic effects is crucial for understanding the mechanism of rice weed suppression [23]. Research has shown that rice cultivars with different allelopathic potentials adopt different weed suppression strategies to cope with weed stress [24]. Here, we addressed rice–weed root interactions by means of root segregation experiments in pot tests that can alter root–root interactions [25]. Under the planting mode of root contact, the root systems of rice and barnyard grass are in complete contact, and the secretions can pass through, simulating the total biological interference of rice with the growth of barnyard grass; and yet the roots of plants are separated but the secretions can pass through by root segregation with nylon mesh, simulating allelopathic effects. The competitive effect between rice and barnyard grass can be calculated by the difference in inhibition rates under the previous two modes. In this study, the enhanced weed inhibitory ability of Le rice induced by ABA was observed through root contact but not through root segregation with nylon (Figure 2), which indicated ABA enhances the inhibitory effect of Le on the growth of barnyard grass mainly due to its impact on the competitive effect of rice roots. This conclusion was further confirmed by the significant increase in the root distribution index (RLD, RSD, RVD, and RWD) of Le in the soil layers after induction (Figure 3). For PI, root segregation with nylon reduced the increment of weed-suppressive ability induced by ABA, but not at a statistically significant level (Figure 2), indicating the induction effect of ABA in PI is more related to root exudates and microbial interactions in the soil, coupled with the weak competition of roots. The results showed highly significant differences in RVD, RWD, RLD and RSD between the control and ABA treatment groups of PI in the 6–12 cm soil circle (Figure 3). He et al. [26] confirmed that PI, with highly allelopathic potential, is dominated by allelopathic effects, while Le relies on resource competition in hydroponic cultivation experiments. The findings in this study further demonstrated that two rice varieties with different allelopathic potentials enhanced their inhibitory effect on the growth of surrounding barnyard grass by adjusting different weed inhibition strategies after ABA induction.

During the interference between rice and barnyard grass, in addition to competing for resources, there are also some other driving factors, namely allelopathic substances secreted by the root system, which are transmitted to the receptor rhizosphere through soil media, thereby inhibiting the growth of surrounding weeds [4]. The synthesis and secretion of allelopathic substances in rice are key factors determining allelopathic potential [14,15]. The eight phenolic acids secreted by rice root in our study are widely recognized as rice allelochemicals, which are mainly classified into benzoic acid derivatives and cinnamic acid derivatives according to their chemical structure [27,28]. In natural conditions, the reversibly adsorbed phenolic acids are available allelochemicals that can be directly utilized by the weeds or microorganisms [29], but most phenolic acids are irreversibly adsorbed to the soil matrix, and the sum of these two types of phenolic acids in soil indicated the total phenolic acid pool [30,31]. In the current study, ABA treatment both significantly elevated the total content of reversibly adsorbed and irreversibly adsorbed phenolic acids in the rhizosphere soil layer of PI (Figure 4), so the ratio of these two types of phenolic acids did not change after induction (Figure 5). This explains that under the two treatment methods of root contact and root segregation, ABA induction increases the weed inhibition rate of PI to a similar extent (Figure 2), because root segregation with nylon mesh does not hinder the spread of rice root exudates from the soil to the weed rhizosphere [32]. However, ABA only caused changes in the total content of irreversibly adsorbed phenolic acids in the rhizosphere soil layer of Le, especially significantly increasing the proportion of the content of irreversibly adsorbed cinnamic acid derivatives in the 6–12 cm soil layer. The result may be related to the changes in the root distribution of Le in the soil layer caused by ABA treatment (Figure 3). Surprisingly, the results obtained in this paper showed no major differences in the phenolic acid contents of PI and Le. In our previous report [20], in response to ABA, two genes (*KSL4* and *CYP75B4*) showed higher expression levels in the allelopathic rice variety than in the non-allelopathic rice variety, which were characterized by the biosynthesis of other allelochemicals, momilactone B and tricin. Hence, we also need to consider other allelochemicals responsible for the allelopathic activity of PI in future study.

### 3.2. ABA Treatment Modulates the Diversity and Community Composition of Bacterial and Microbial Taxa in the Rhizosphere Soil of Allelopathic and Non-Allelopathic Rice

PI and Le via ABA induction recruited different unique and core bacteria and microbes to the rhizosphere with significant differences in relative abundances (Figure 6, Figures S1 and S2). In particular, a higher diversity of rhizosphere microbiota associated with PI than that in Le was observed (Figure 6A), which was speculated to be related to the increased secretion of secondary metabolites in the rhizosphere of allelopathic rice [11,13,14]. ABA treatment significantly affected the composition of core and unique microbiomes (Figure 6C). Especially, the relative abundances of bacterial *Myxococcales* and *Pedosphaerales* in the rhizosphere soil of rice PI were higher than in the uninduced soil. It was reported that *Pedosphaerales* belonging to the phylum *Verrucomicrobiota* were correlated positively to metal concentrations (Cr, Mg, Zn, etc.) in forested soil [33,34]. In addition, Fang et al. [14] reported that the population of *Myxococcus* species (*M. xanthus*) was significantly higher in the PI/barnyard grass system than in the Le/barnyard grass system, which was promoted by the phenolic acids secreted from rice root tissue in the hydroponic system. Although the relative abundance of this *Myxococcales* in the rhizosphere soil of PI and Le rice was elevated compared to the uninduced group, the degree of increase in the rhizosphere soil of PI was higher than its level in Le (Figure 6C). In addition, the relative abundances of bacterial *Burkholderiales* in the rhizosphere soil of Le were higher than that in the uninduced soil and that of PI. Several *Burkholderia* species, considered as beneficial bacteria in the natural environment, can be used as biocontrol agents for phytopathogenic fungi and are able to enhance plant growth [35,36].

The chemical composition of different root exudates and microbial substrate preferences propel the assembly mode of rhizosphere microbial communities [37]. Different constituents and concentrations of in the root exudates from different allelopathic rice varieties may result in different regulatory effects with specific microorganisms that can affect the interaction with rice. In the current study, the core microbial changes had different trends in the rhizosphere of PI and Le in response to ABA application. The present study revealed that higher concentrations of reversibly adsorbed phenolic acids recruited more bacteria of *Anaerolineales*, *Bacteroidales*, and *Myxococcale* in the rhizosphere of PI induced by ABA (Figure 7A). Intriguingly, it was found that the *OsPAL2-1*-overexpressed transgenic line (PO) had significantly greater abundance of Myxococcus species (*M. xanthus*) compared with PI, which was related to the process involved in the influence of the *OsPAL2-1* gene expression abundance on the synthesis and secretion of phenolic acids, leading to proliferation of the myxobacteria in rhizosphere soil which may produce the potential allelochemical quercetin [13]. In other studies, protoporphyrinogen oxidase (PPO) derived from the soil bacterium *Myxococcus xanthus* has been considered as a perfect target for the development of new herbicides, and transgenic rice overexpressing *PPO* showed significantly improved drought tolerance [38,39,40]. The relationships suggested that higher concentrations of reversibly adsorbed phenolic acids in rhizosphere soil of allelopathic rice induced by ABA play important roles in the chemotactic aggregation of the special microbe *Myxococcale* in the rhizosphere soil, which may strengthen allelopathic effects. It would be valuable to investigate the direct research evidence of the regulatory mechanism between specific *Myxococcale* species and active phenolic compounds in future experimental manipulations.

However, for non-allelopathic rice Le, ABA only caused changes in the total content of irreversibly adsorbed phenolic acids in the rhizosphere soil layer, which recruited more bacteria of *Sphingomonadales*, *Cyanobacteriales*, *Polyangiales,* and *Rhizobiales* (Figure 7B). In the light of relevant research, *Cyanobacteria* are increasingly applied as biofertilizers for improving soil and crop productivity, and this has been demonstrated in rice-growing areas where the most efficient nitrogen-fixing cyanobacteria are present in paddies [41]. Many species of *Sphingomonas* have been shown to promote plant growth and enhance plant stress resistance, which are believed to be due to their functions such as nitrogen fixation, phosphate solubilization, and production of plant growth hormones [42]. More specifically, Dao et al. [43] reported that *Rhizobiales,* enriched in the rhizosphere of drought-resistant sugarcane varieties, were positively correlated with root tip number and total root length, increasing the distribution area of roots and, thus, improving water and nutrient uptake by the roots. We can speculate that the predominantly enhanced *Rhizobiales* in the rhizosphere soil of Le are due to the increased recruitment of soil carbon sources (inactive reversibly adsorbed phenolic acids) after ABA treatment, which together enhance the competitive effect of its root system in the soil.

## 4. Materials and Methods

Two rice cultivars (PI and Le) and one barnyard grass biotype were used in this study. The allelopathic rice accession PI and non-allelopathic rice accession Le have been internationally recognized [44]. Mature susceptible barnyard grass (*Echinochloa crus-galli*) seeds were collected from rice fields the year prior to the experiment and stored in a refrigerator for over 1 year. Soil was collected randomly from a paddy field. The soil was silt loam of pH 6.47, with 247 mg kg^−1^ organic matter, 629 mg kg^−1^ total N, 50.8 mg kg^−1^ available P, and126 mg kg^−1^ available K. Soil samples were air-dried, mixed, and used for the culture experiments after residual plant roots and branches were removed.

### 4.1. Pot-Culture Experiments of Rice–Barnyard Grass Mix

The first experiment investigated the performance of paddy weeds in mixed culture with PI and Le after exogenous ABA induction. In order to visually and effectively observe the weed-control activity, the established inhibitory-circle method described in our previous study was applied [6]. A total of 24 plastic pots (30 cm diameter × 16 cm height) containing 30 kg paddy soil were watered with 10 L of water, mixed well, and kept stationary for 1 day. Five pre-germinated seeds of both PI and Le exhibiting uniform growth were planted in the central area (6 cm diameter) of each pot, watered daily, and paddy weeds were removed manually during the experimental period. When the rice seedlings grew to the 3-leaf stage, five pre-germinated seeds of barnyard grass were planted uniformly in a circle around the rice seedlings at 12 cm apart from the base of the rice seedlings according to a previous report [30]. Monocultures of five barnyard grass seeds without rice seedlings served as controls. A 100 mL aliquot of ABA solution at the concentration of 3, 9, and 12 μmol/L was slowly added to each rice root system, guaranteeing each of the roots were adequately exposed to exogenous ABA, and the induction was repeated after 1 week according to the procedure of Li et al. [20]. At the same time, ABA treatment was also conducted in the same soil area of the monoculture of barnyard grass. The experiments were conducted in a completely randomized design with four replicates for each treatment. Pots were placed in the greenhouse in 25–37 °C night and daytime temperatures and 45–80% relative humidity maintained during the growing season in 2024, and watered daily and randomized once a week. Two weeks after induction, plant heights of barnyard grasses in each group were measured. The above-ground parts of barnyard grasses in each pot were harvested to record their measured fresh weight and dry weight after freeze-drying for at least 48 h. To assess the weed inhibition activity of the rice cultivars, the inhibition rate was calculated as follows: inhibition % = (1 − T/C) × 100, based on the plant height, fresh weight, and dry weight of the barnyard grass co-cultured with the two rice cultivars (T) and the monoculture barnyard grass in controls (C) [28].

The second experiment was conducted to evaluate the impact of exogenous ABA treatments on the root competition effect and allelopathic effect of PI and Le. A series of 30 cm (diameter) × 16 cm (height) plastic pots were divided into two root treatments for the growth of rice in mixed culture with barnyard grasses for each variety, with modification of the method described by Ding et al. [32]. The pots in the first group (root-contact group) had full contact between rice and barnyard grass, which could simulate interference of the two rice cultivars with the paddy weeds. In the second group (root-segregation group), the pots contained a vacant cylinder (12 cm diameter, 15 cm height) in the central area, while the cylinders were covered with 30 μm nylon mesh. The 30 μm nylon mesh prevented penetration by the root systems but allowed chemical and microbial interactions in the pots, which could simulate the allelopathic effect of rice on the weed growth. Based on the results of Experiment 1, we selected an appropriate ABA concentration of 9 μmol/L for exogenous induction of each rice cultivar under two root treatments, similarly to Experiment 1 as described above. Monocultures of barnyard grass in a pot, for each group with or without root segregation, served as controls. The experiments were conducted in a completely randomized design, with three replicates for each treatment. After 2 weeks of ABA induction, the aboveground parts of the barnyard grass were collected, and the plant heights and dry weight of the barnyard grasses were measured according to Experiment 1.

### 4.2. Measurement of Lateral Distribution of Rice Roots in Different Soil Layers

The pot-culture experiments with two rice cultivars in mixed culture with barnyard grasses were conducted as described in Experiment 1, with 9 μmol L^−1^ ABA induction. The experiments were conducted in a completely randomized design, with three replicates for each treatment. Two weeks after induction, the above-ground parts of each rice cultivar were cut. All the roots of the different rice cultivars were sampled in the 0–5 cm soil depth and collected in horizontal distance intervals of 0–6 and 6–12 cm from the center area of the rice seedlings. The lateral distribution of the rice roots was determined according to the procedure of Li et al. [6]. Briefly, all soil samples were soaked in water and then washed with a metal-sieve stack (pore size of 0.40 mm), and all roots were manually collected. Clean roots were immediately scanned by an Epson Expression 11000XL scanner (Seiko Epson Co., Ltd., Nagano-ken, Japan) to yield a grayscale image. Root length (cm), root surface area (cm^2^), and root volume (cm^3^) were determined using the WinRHIZO Pro software (Regent Instruments Inc., Quebec, QC, Canada). After the analysis, root samples were freeze-dried for at least 48 h to measure the root dry weight. Then, we calculated root length density (RLD), root surface area density (RSD), root volume density (RVD), and root dry weight density (RWD) for different root samples, which were calculated as follows: RLD = *L*/*v* (cm cm^−3^), RSD = *S*/*v* (cm^2^ cm^−3^), RVD = *V*/*v* (cm^3^ cm^−3^), and RWD = *W*/*v* (mg cm^−3^), where L is root length, S is root surface area, V is root volume, W is root dry weight in each soil layer, and v is the volume of the core soil sampled in each layer.

### 4.3. Quantification of Phenolic Acids in Different Soil Layers

Two weeks after 9 μmol L^−1^ ABA induction in pot-culture experiments as described in Experiment 1, the soil samples of the two rice cultivars with or without ABA induction were sampled as described in Section 4.3. The experiments were conducted in a completely randomized design, with three replicates for each treatment. The samples from the same rice cultivar were mixed evenly, slightly air-dried, and sieved (<5 mm) after removing the plant fragments, and prepared for further extraction of phenolic acids.

Phenolic acids in the soil can be divided into reversibly adsorbed phenolic acids and irreversibly adsorbed phenolic acids, which can be extracted from soils using different solvents according to Blum [29] and Li et al. [30]. Three replicates (100 g) of each soil sample above were placed into 250 mL conical flasks and treated separately as follows: (a) 100 mL of 0.25 mol L^−1^ sodium citrate (pH = 7.0) was added to the flask, which was then shaken for 2.5 h and centrifuged at 11,000 rpm for 10 min. The combined supernatant was filtered using a 0.45 μm filter, and phenolic acids that reversibly adsorbed to the soil were extracted, known as “reversibly adsorbed phenolic acids”; (b) 100 mL 0.5 mol L^−1^ NaOH was added to the remaining soil samples of treatment a, followed by shaking again for 2.5 h. The pH was adjusted to 2.5 with HCl, and the soil sample was centrifuged at 11,000 rpm for 10 min to remove the humic acid. The supernatant was filtered using a 0.45 μm filter, the pH was adjusted to 5.0 using NaOH, and the phenolic acids that irreversibly adsorbed to the soil were extracted, known as “irreversibly adsorbed phenolic acids”. Solid phase extraction and high-performance liquid chromatography (HPLC) were conducted to quantify the content of single phenolic acids in different soil layers as described previously [6]. The concentrations of single phenolic acids in each soil sample were quantified by interpolating the peak area on the HPLC chromatogram to a standard curve constructed from the peak area of eight authentic phenolic acids (protocatechuic, *p*-hydroxybenzoic, vanillic, syringic, *p*-coumaric, ferulic, salicylic, and cinnamic acid).

### 4.4. Rhizosphere Soil DNA Extraction and Sequencing in Different Soil Layers

The pot-culture experiments of the two rice cultivars in mixed culture with barnyard grasses were conducted as described in Experiment 1 with 9 μmol L^−1^ ABA induction. The experiments were conducted in a completely randomized design, with three replicates for each treatment. Two weeks after induction, the rhizosphere soil samples of the two rice cultivars and monoculture of barnyard grass with or without ABA induction were carefully collected. Total DNA was extracted from 0.5 g rhizosphere soil of each sample, using a DNA Extraction Kit for soil according to the manufacturer’s instructions (E.Z.N.A.^®^ Soil DNA Kit, Omega Bio-Tek, Norcross, GA, USA). The quantity and quality of DNA were assessed using a NanoDrop ND-1000 Spectrophotometer (Thermo Fisher Scientific, Waltham, MA, USA) and on 0.8% agarose gel electrophoresis, respectively. The V3-V4 region of the bacterial 16S rRNA gene was united with adapter sequences and barcode sequences by amplification with the universal barcoded primers 338F/806R. PCR amplification was performed, and the products were purified, quantified, and sequenced on an Illumina Novaseq platform (San Diego, CA, USA). Raw sequences were sorted based on the unique sample barcodes and denoised using the QIIME pipeline. High-quality sequences were then clustered in operational taxonomic units (OTUs) at a minimum of 97% identity in the SILVA database (version 132). The unclassified OTUs and the reads identified as chimeric sequences were removed using the Vsearch algorithm (version 2.3.4).

### 4.5. Statistical Analysis

Microbial community analysis was performed using BMK Cloud (www.biocloud.net, accessed on 5 May 2024). The Alpha diversity (Shannon index) calculation was performed using QIIME2 (https://qiime2.org/, accessed on 5 May 2024) and R software (version 4.4.3). Beta diversity was determined by QIIME, then evaluating the degree of similarity of microbial communities from different samples. The beta diversity was analyzed by principal coordinate analysis (PCoA) based on Bray–Curtis distance. The Spearman correlation analysis between microbial communities and phenolic acid contents in soils were analyzed using R software. All quantitative data are expressed as the means ± SEs, and the differences in traits among plant groups (i.e., plant growth, root distribution, and phenolic acids) were tested by analysis of *t*-test or variance (ANOVA) using the SPSS 22.0 software (IBM Corp., Armonk, NY, USA).

## 5. Conclusions

In summary, this work revealed the induction mechanism of exogenous ABA on the weed inhibition strategies of allelopathic and non-allelopathic rice accessions underground. Using two pot experiments through an inhibitory-circle method under root contact or root segregation with nylon mesh, we found that the two rice varieties with different allelopathic potentials enhance their inhibitory effect on the growth of surrounding barnyard grass by adjusting their different weed inhibition strategies after ABA induction. The compelling evidence and mechanisms involved were as follows: (i) ABA enhances the inhibitory effect of non-allelopathic rice Le on the growth of barnyard grass mainly due to its impact on the competitive effect of rice roots, with a significant increase in the root distribution of Le rice in soil layers after induction; (ii) the induction effect of ABA in allelopathic rice PI is more related to root exudates in the soil, which was proved by the results of ABA treatment, which significantly elevated the total content of both reversibly adsorbed and irreversibly adsorbed phenolic acids in the rhizosphere soil layer of PI; (iii) exogenous ABA could change the bacterial composition in the rhizosphere soil of two rice accessions, with the change in the species composition in the rhizosphere soil of PI being greater. The different constituents and concentrations in the root exudates from these two rice varieties may result in different regulatory effects on specific microorganisms in the rhizosphere soil after ABA induction. However, such responsiveness needs to be explored in other rice varieties using field applications to determine whether this is a general phenomenon.

## Data Availability

All data supporting the findings of this research are available within the paper. Raw data used here can be obtained directly from the authors.

## Supplementary Materials

The following supporting information can be downloaded at: https://www.mdpi.com/article/10.3390/plants14182813/s1, Figure S1: The overlap of taxonomical operational taxonomic units (OTUs) in the soil layers of PI and Le induced by exogenous ABA; Figure S2: Phyla distribution of the rhizosphere microbiota compositions of two soil layers of PI and Le induced by exogenous ABA.
