# Supplementary material for: Effects of Abscisic Acid Induction on the Underground Weed Inhibition Strategies of Allelopathic and Non-Allelopathic Rice Accessions
Source: Plants (Basel). 2025 Sep 9;14(18):2813. doi: 10.3390/plants14182813 (PMC12473845; doi:10.3390/plants14182813)
Supplement: Supplementary file 1 [file plants-14-02813-s001.zip › plants-3823845-supplementary.pdf]

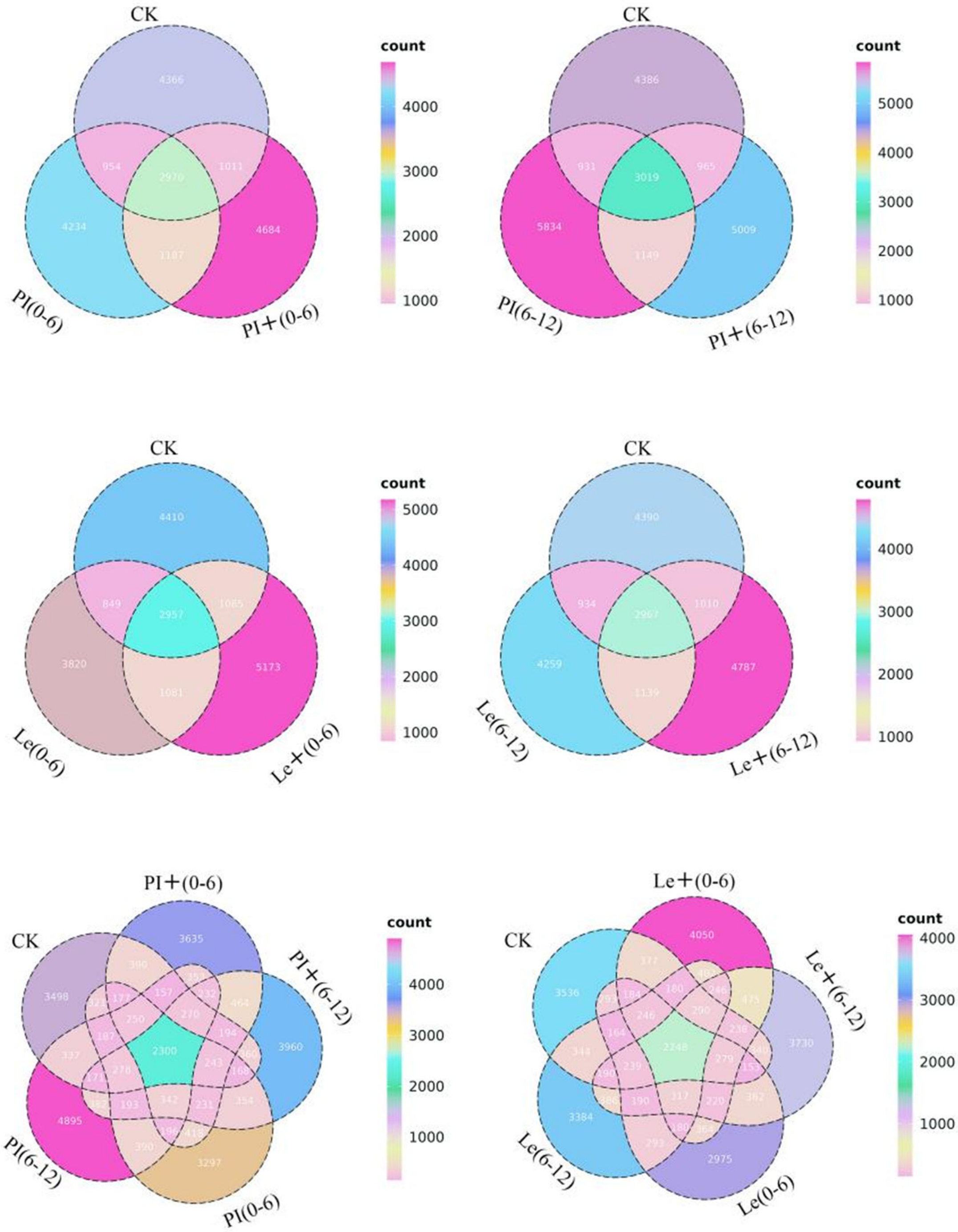

Figure S1: The overlap of taxonomical operational taxonomic units (OTUs) in the 0-6 and 6-12 soil layers of PI and Le induced by exogenous ABA.

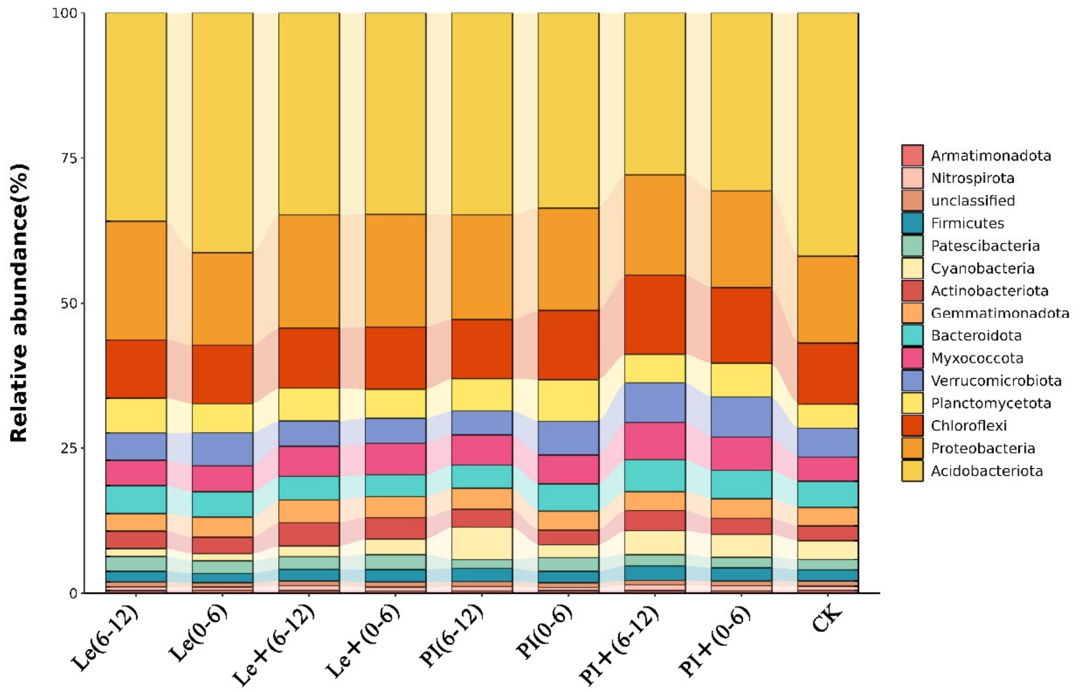

Figure S2: Phyla distribution of the rhizosphere microbiota compositions of two soil layers of PI and Le induced by exogenous ABA.
